# Notes and News

**Published:** 1895-01-26

**Authors:** 


### NOTES AND NEWS.

COLLECTED AND COMMUNICATED.

SMALL-POX shows no signs of abatement in Dublin as yet.

STEPS are being taken towards the organisation of the twelfth International Congress of Medicine at Moscow in 1897. Matters being thus well in hand, the mistakes visible at previous congresses should be obviated.

WHILST the medical profession in the older countries is suffering from increasing overcrowding, it is stated that in Australia there is still a good opening for medical men. The outlook for the specialist is moreover especially promising.

IT must be a source of gratification to all Dr. Cullingworth's friends that his colleagues at St. Thomas's have shown their sympathy with him on the occasion of a vexatious action brought against him by a litigious patient, by the presentation of a cheque at the annual dinner of the staff, held last week.

THE following appointments were made at the Chelsea Hospital for Women, on the 16th inst., by the Board of Managers: Drs. W. Duncan and W. H. Fenton, physicians to the in-patients; Robert O'Callaghan, F.R.C.S.I., surgeon to the in-patients; Drs. J. Inglis Parsons, A. E. Giles, and T. Eden, physicians to the out-patients; Dr. E. J. Maclean, pathologist, and G. H. Berkeley, M.B., B.C., M.R.C.S., registrar.

WE are glad to see in the columns of the *Oxford Journal* that the paper admits the responsibility of the University of Oxford towards the Radcliffe Infirmary, and also that the University has not acted up to that responsibility. The *Oxford Journal* says: "Whilst the efforts of many distinguished University residents are praiseworthy, the indifference of the University is a painful fact. If THE HOSPITAL can make any impression on the Hebdomadal Council and Congregation, and convince them that the interest as well as the duty of the University lies in joining the City and county, in order to make the infirmary worthy of all three, we shall sincerely rejoice."

IN France advertising has reached such a point that the name of a medical specialist appears on the curtain of a theatre, together with the hours and address at which he can be seen.

SPEAKING at a meeting of the New York Academy of Medicine, Dr. Winters condemned the use of a tent in diphtheria, contending that the close atmosphere was more harmful than the medicated inhalation was beneficial.

THE Right Hon. the Lord Chancellor has kindly consented to preside at the Festival Dinner, in aid of the funds of the Royal Sea-Bathing Infirmary for Scrofula, on Wednesday, February 20th next, at the Whitehall Rooms, Hotel Metropôle, S.W.

LORD KIMBERLEY has been memorialised on behalf of British shipowners trading with the Levant. Hospital dues are levied on British ships at Constantinople and Smyrna, and have now reached a surplus of £16,000. It is therefore contended that a fresh and more suitable arrangement should be made.

LIVERPOOL was most unfortunate in the weather prevailing on its "Hospital Sunday." It is not to be wondered at that the collections in nearly all cases were smaller than those of last year. The full list is not yet forthcoming, but it is to be hoped that the total will not show a diminution. as those unable to attend the services will have opportunity of forwarding their intended contributions to the authorities.

AT a special meeting of the board of delegates of the Metropolitan Hospital Saturday Fund on Saturday evening, it was agreed to distribute a sum of £17,600 to be divided among 165 participating institutions as follows, viz.: Thirty general hospitals, £6,201 13s.; 63 special hospitals, £6,392 1s.; 35 dispensaries, £1,062 9s.; 17 convalescent homes, £1,496 19s.; and 18 miscellaneous institutions, £2,105 17s. The following special resolutions were passed by the board, namely: "Queen's Jubilee Hospital.—The Distribution Committee recommend that a sum be set aside equal to a grant to the Queen's Jubilee Hospital on the usual plan of award; that this sum—viz., £63 13s.—be paid to the hospital when the serious charges made in the Press have been met and publicly refuted to the satisfaction of your board; and when the following appointments have been made by the governors of the institution, viz., treasurer, finance committee, house committee of not less than six members meeting weekly." "Women's Hospital, Chelsea.—The Distribution Committee having carefully considered the official report of the committee of inquiry, are of opinion that a lamentable condition of affairs for the year 1893 has been disclosed. They hesitate to recommend the removal of this institution from the list, as they hope and believe great efforts are being, and will be made, to restore and justify public confidence. The committee recommend a grant being made in accordance with the plan of award, but that the amount, viz., £95 1s., be not paid over until the authorities of the hospital have fully satisfied your board that the requirements and reforms mentioned in the official report have been carried out. Furthermore, your committee strongly approve the action of Dr. Louis Parkes, Medical Officer of Health, in calling public attention to the matter."

